# A New Instrument for the Administration of Compressed Air

**Published:** 1877-12

**Authors:** 


### A New Instrument for the Administration of Compressed Air.

Dr. F. H. Davis, of Chicago, has devised a new instrument that is designed to be a cheap substitute for the more expensive ones, such as Waldenburg's, now in use. It consists of a small gasometer, fifteen inches in diameter by twenty-four inches in height, and should be made of galvanized iron. On the side of the outer tank two upright bands are soldered, embracing between them a slip of wood that reaches twelve inches above the tank, and to the extremity of which is hinged a wooden lever two feet long. A handle in the middle of the inner tank is attached to the middle of the wooden lever. But slight exertion on the part of the patient in raising or lowering this lever, produces all the pressure or suction necessary. The apparatus can be made by any tinsmith, and with three feet of three-quarter inch hose, costs from four to five dollars. The patient inspires the compressed air, or, *vice versa*, expires into rarefied air and inspires again the ordinary atmosphere. To obtain more marked result two gasometers are sometimes employed—one to inspire from, and one to expire into. As the lever is raised the patient expires into the one, the other being at the same time raised full of fresh air; he then inspires the fresh air as the lever is depressed, the foul air at the same time emptying itself from the first tank. One mouthpiece only need be used, and a light guage may be attached to show the exact amount of pressure or suction. Fitted out in this way the apparatus would cost from fifteen to twenty dollars. Dr. Davis has seen the advantage of this treatment in several classes of cases. It has been chiefly in those young persons of slight physique, whose chests were flat and narrow, and had deficient expansive power. While physicial examination showed no tubercular deposits, there was a sensitive condition of the air-passages causing frequent attacks of cold. In such persons the inhalation of compressed air from five to ten minutes, once or twice a day, produced very decided improvement. Under its use, in combination with some tonic, such as the malt extract or cod-liver oil, the patient's condition improved, his carriage became more erect, inspiration deepened, and the sensitiveness to catarrhal attacks lessened. Where there is incipient or primary tuberculosis, such as is shown by a loss of flesh and strength, with diminished appetite, shortness of breath on exercise, slight pains in the chest, usually a hacking cough and expectoration of a mucous character, rather harsh bronchial respiration, and more or less dulness on percussion, it is thought that the question of improvement under compressed air will depend a good deal upon the hereditary taint. When the phthisis is *inflammatory* in its origin, the best of results may be anticipated, unless the stage of softening has commenced, and then this treatment should not be attempted for fear of hemorrhage. In emphysema and valvular diseases of the heart the author has had no experience.—*Chicago Med. Journal & Examiner*, Oct., 1877. *Medical Record.*

